# Case report: A case of fetal umbilical vein varix presenting disseminated intravascular coagulation, polycythemia, and neonatal hepatitis in an extremely low birth weight infant

**DOI:** 10.3389/fped.2023.1154820

**Published:** 2023-03-30

**Authors:** Mariko Sekiguchi, Takeo Mukai, Yoshihiko Shitara, Kohei Kashima, Takahiro Seyama, Keiichi Kumasawa, Naoto Takahashi

**Affiliations:** ^1^ Department of Pediatrics, The University of Tokyo Hospital, Tokyo, Japan; ^2^ Department of Obstetrics and Gynecology, The University of Tokyo Hospital, Tokyo, Japan

**Keywords:** umbilical vein varix, extremely low birth infants, polycytemia, neonatal hepatitis, disseminated intravascular coagulation, fetal thrombotic vasculopathy

## Abstract

Reports on the clinical course of fetal umbilical vein varix in premature infants are limited. We report a case of an extremely low body weight infant with intra-abdominal umbilical vein varix who developed disseminated intravascular coagulation, polycythemia, and hyperbilirubinemia after birth; late-onset neonatal hepatitis; and fetal thrombotic vasculopathy confirmed by placental histopathology. Ultrasonography after birth showed a dilated portion of the umbilical vein at the hepatic hilum with thrombi inside. We speculate that the umbilical vein varix caused the fetal thrombotic vasculopathy, and the presence of umbilical vein varix and fetal thrombotic vasculopathy in combination with prematurity caused coagulopathy, polycythemia, hyperbilirubinemia, and hepatitis. Despite the favorable outcomes reported in the literature, premature infants with umbilical vein varix may require careful observation and management for coagulopathy and late-onset hepatitis. Furthermore, placental histopathology could aid in the understanding of various clinical outcomes in infants with umbilical vein varices.

## 1. Introduction

The umbilical vein varix (UVV) is a focal dilation on the umbilical cord vein. Varicose dilation of an umbilical vessel is a rare anomaly that is diagnosed prenatally by identifying an anechoic cystic mass between the fetal abdominal wall and inferior portion of the liver (1–3). No criteria for UVV diagnosis have been established, but some authors suggest that an intra-abdominal transverse diameter 1.5 times greater than the diameter of the intrahepatic umbilical vein can be considered diagnostic (4). Others consider the diagnosis of UVV if the varix diameter is greater than 9 mm, at least 50% wider, or is 1.5 greater than the nondilated portion of the umbilical vein (1, 5). The incidence of UVV is estimated to be 0.4–1.1 in 1,000 live births, and recent reports indicate favorable perinatal outcomes of UVV without congenital anomalies and chromosomal abnormalities (1, 5). The majority of the literature on UVV focuses on prenatal diagnosis and management, and only a few report on its neonatal clinical course. Herein, we present a case of an extremely low birth weight infant (ELBWI) presenting with disseminated intravascular coagulation (DIC), polycythemia, and neonatal hepatitis following fetal intra-abdominal UVV.

## 2. Case presentation

**Figure 1:**
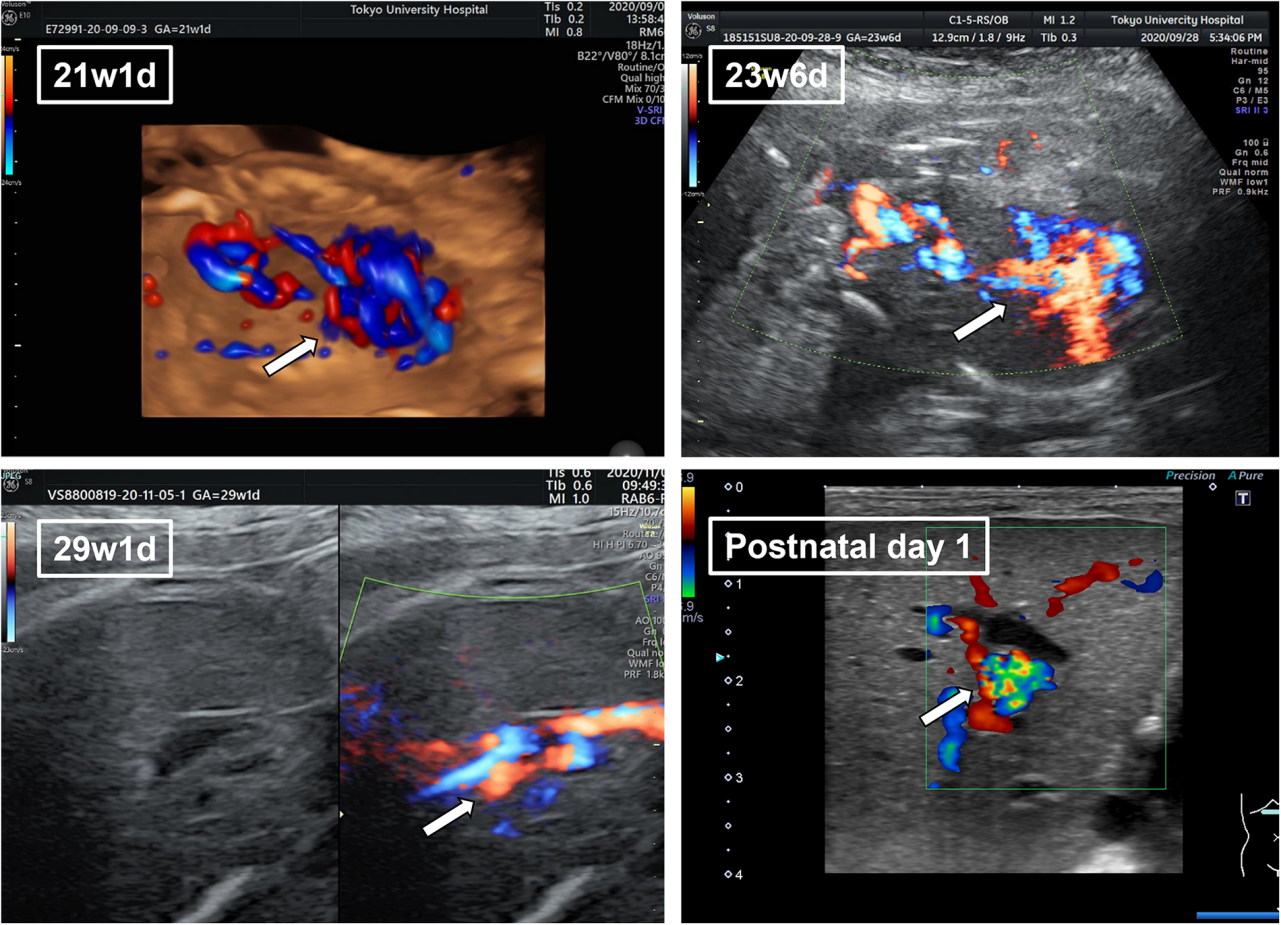
The fetal ultrasonography of umbilical vein varix (UVV) in the perinatal period. Fetal ultrasonography revealed a 17 mm × 10 mm UVV near the hepatic hilum (arrow). Dilation of the umbilical vein remained the same throughout the perinatal period.

The patient’s mother was 37-year-old primigravida with no history of infection during her pregnancy. The pregnancy was uneventful until the dilation of the umbilical vessel of the fetus was noted at the routine clinical visit at 21 weeks of gestation. The patient was referred to our hospital in the same week. A 17 mm × 10 mm UVV near the hepatic hilum was detected on the first ultrasonography performed in our hospital. No congenital abnormalities were detected in the heart or brain. The mother was followed up every 3–4 days, and no change in the dilation of the umbilical vein was observed during follow-up (Figure 1). At 28 weeks of gestation, the mother developed hypertension and was admitted to our gynecology department. On admission, fetal brain-sparing and fetal growth restriction (FGR) occurred. At 29 weeks and 2 days of gestation, maternal inflammation worsened as the serum C-reactive protein level increased. The fetus was diagnosed with non-reassuring fetal status, and an emergency cesarean section was performed.

At birth, he was groaning and had respiratory retractions. His APGAR scores were 3, 6, and 8 at 1, 5, and 10 min, respectively. He was diagnosed with respiratory distress syndrome (RDS) and was placed on continuous positive airway pressure (CPAP) followed by tracheal intubation. He was small for gestational age (SGA), with a weight of 934 g (1.7 percentile), length of 39 cm (14.2 percentile), and head circumference of 26 cm (34.7 percentile). Ultrasonography on admission revealed no intraventricular hemorrhage or congenital heart disease. Abdominal ultrasonography revealed remnants of the ductus venosus, and the umbilical portion was dilated with a floating object and a hyperechoic region inside (Supplementary Video S1). The flow volume at the origin of the ductus venosus significantly increased, and an influx into the inferior vena cava was observed. Laboratory examinations revealed coagulopathy, polycythemia, and hyperbilirubinemia. The prothrombin time (PT) and activated prothrombin time (APTT) were unmeasurable, D-dimer at 4.8 ug/ml, hemoglobin (Hb) of 24.8 g/dl, hematocrit of 71.1%, reticulocytes of 6.0%, platelet count of 11,000/µl, aspartate aminotransferase (AST) of 83 IU/L, alanine aminotransferase (ALT) of 13 IU/L, lactose dehydrogenase (LD) of 1,227 IU/L, total bilirubin of 7.1 mg/dl, and direct bilirubin of 0.9 mg/dl. The infant’s blood type was A Rh D (+) and that of the mother was AB Rh D (+) resulting in ABO and Rh D incompatibility. All serological tests for TORCH syndrome on admission were negative.

**Figure 2:**
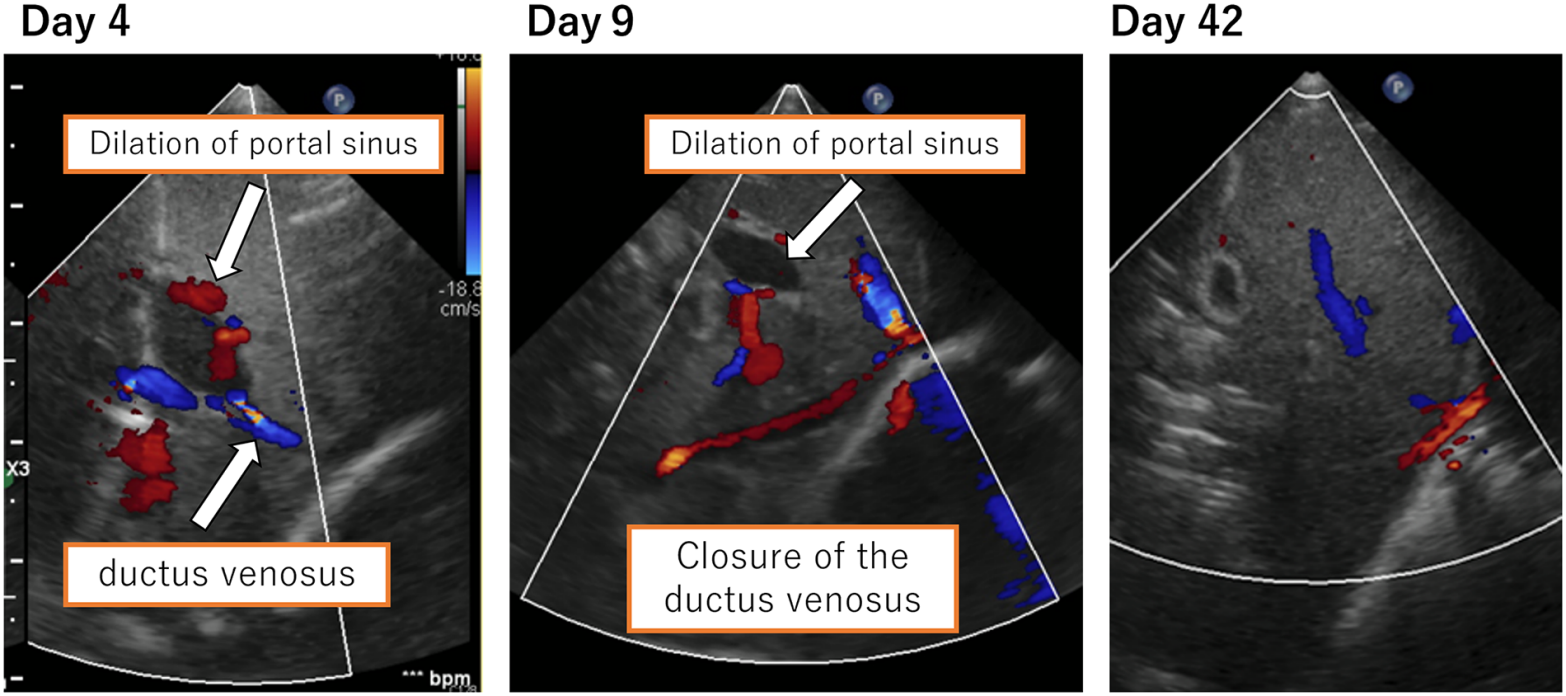
Ductus venosus and dilation of the portal sinus. Closure of the ductus venosus was confirmed on the 9th day of life. Dilation of the portal vein resolved on the 42nd day of life.

**Figure 3:**
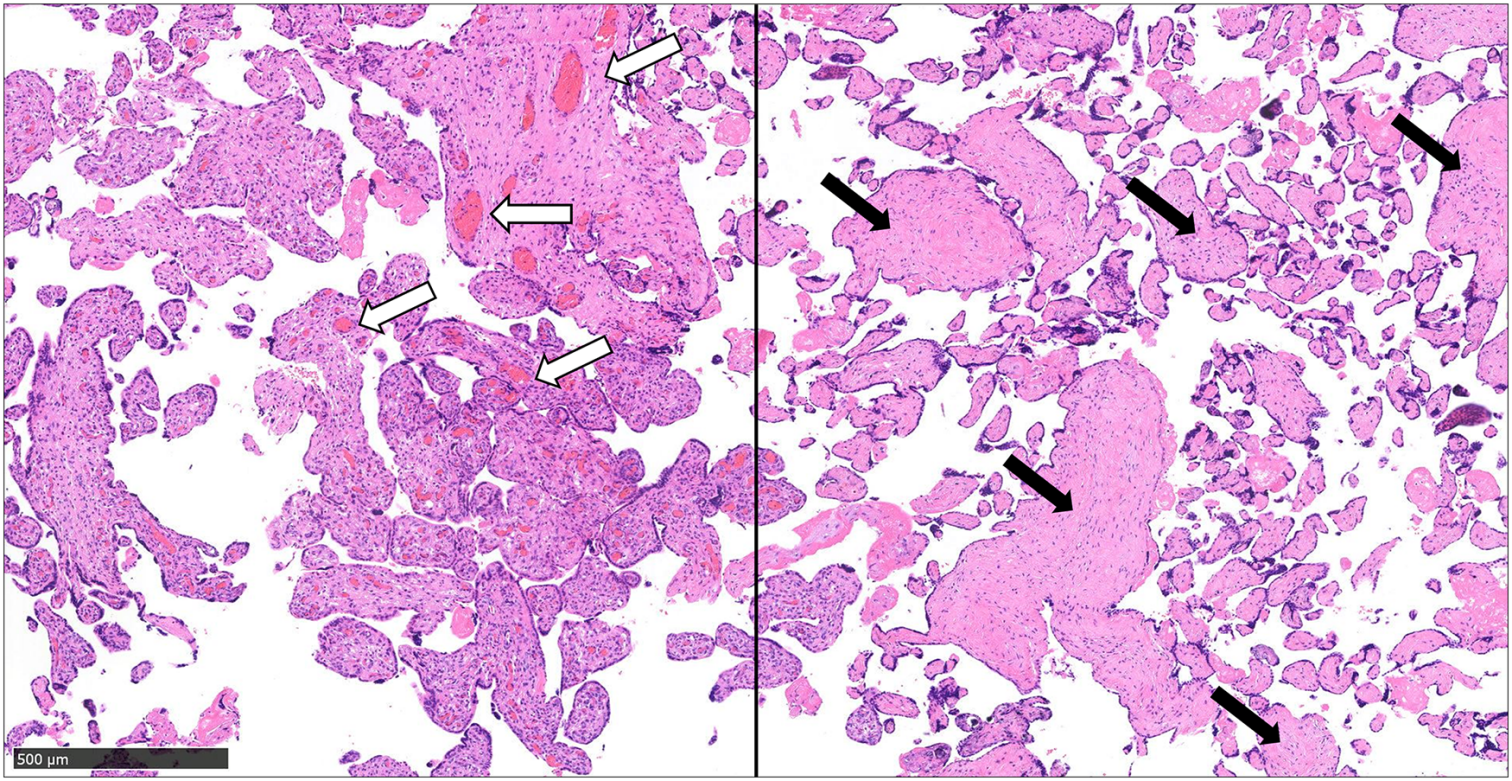
The pathological diagnosis of the placenta indicated fetal thrombotic vasculopathy. Pathological findings of the placenta, in addition to normal capillaries (white arrows), thrombus formation in the umbilical artery, and reduction and collapse of villous interstitial capillaries in the periphery (black arrows) are observed (scale bar = 500 µm).

We suspected DIC and neonatal jaundice; thus, transfusions of albumin, fresh frozen plasma (FFP), and platelet concentrate as well as partial exchange transfusion were performed within 5 h of birth. A single transfusion resulted in a decrease in Hb to 15.4 mg/dl and an increase in platelet count on first day of life; therefore, no additional transfusions were indicated. For neonatal jaundice, phototherapy was continued until the 8th day of life. On the second day of life, the flow of the patent ductus arteriosus (PDA) shunt increased. With the shunt from patent ductus venosus (PDV), the cardiac load increased with brain natriuretic peptide (BNP) measured at 1,897 pg/ml. We thus administered ibuprofen on the second day of life, which reduced the PDA flow and completely closed it on the 6th day of life. We confirmed the closure of the ductus venosus on the 9th day of life, but the dilated portal vein persisted. On the 42nd day of life, dilation of the portal vein resolved (Figure 2). The pathological diagnosis of the placenta indicated fetal thrombotic vasculopathy (FTV), but no chorioamnionitis or umbilical cord inflammation was found (Figure 3).

On the 34th day of life, a significant elevation of liver enzymes was observed with no other signs and symptoms. AST and ALT levels increased to 200 and 47 IU/L, respectively. Hepatitis B virus and Epstein-Barr virus serologies were negative. We examined the serologies for TORCH syndrome including cytomegalovirus DNA in urine for second time, and all tests were negative. Biliary scintigraphy revealed no signs of biliary atresia. Neonatal mass screening for inborn errors of metabolism ruled out citrullinemia and galactosemia. Conversely, biomarkers for liver fibrosis such as hyaluronic acid, type IV collagen, and total bile acid were elevated at 358.7 ng/ml, 32.1 ng/ml, and 126.9 μmol/L, respectively. Thus, the patient was diagnosed with neonatal hepatitis. Ursodeoxycholic acid was administered on the 46th day of life, hepatic enzymes gradually decreased, and fibrosis markers decreased on the 127th day of life.

The patient was started on enteral nutrition on the 6th day of life and was shifted to autonomous feeding on the 66th day of life. Due to the development of aggressive posterior retinopathy of prematurity (ROP) on the 36th day of life, anti-vascular endothelial growth factor inhibitors were administered intraocularly, and laser photocoagulation was performed on the 43rd day of life.

As for neurological testing, brain magnetic resonance imaging on the 87th day of life revealed no signs of microthrombus, and electroencephalography was normal. Feeding significantly improved and he was discharged on the 132nd day of life (corrected age of 48 weeks and 1 day) with a weight of 3,641 g, length of 53.3 cm, and head circumference of 37 cm. No neurodevelopmental disorders have been reported during follow-ups up to the present day of 2 years and 2 months.

This study was approved by the Institutional Review Board of the Ethics Committee of the University of Tokyo Hospital (approval ID: 2701).

## 3. Discussion

To the best of our knowledge, this is the first reported case of UVV in ELBWI with DIC, polycythemia, and neonatal hepatitis. In our case, the patient had DIC requiring FFP and platelet transfusion, polycythemia requiring partial exchange transfusion, and transient neonatal hepatitis. Despite extensive treatment during the first few hours of life, there was no development of intraventricular hemorrhage or cranial thrombosis.

UVV is typically detected in prenatal ultrasound studies and requires careful observation during pregnancy. Although the etiology of UVV is not fully understood, it is considered a developmental lesion rather than a congenital anomaly since UVV is detected after 20 weeks of gestation. Initial reports on UVV showed an association between FGR and intrauterine fetal death (IUFD). However, recent studies have suggested that UVV without chromosomal and structural anomalies, often referred to as isolated UVV, yields insignificant association with FGR, IUFD, and other complications (1, 3, 4). Although the current case was considered to be that of an isolated UVV, there were severe neonatal complications. To understand the pathophysiology of our case, several disease entities, specifically UVV, ELBWI, and FTV, should be considered.

Thrombosis is one of the complications of UVV during pregnancy. Thrombus formation in a varix can be detected by ultrasound during follow-up. Once a thrombus is detected, termination of pregnancy may be an option with good neonatal outcomes and absence of coagulopathy (6–8). Despite increased vulnerability to thrombosis due to UVV during pregnancy, coagulopathy and DIC seem rare. Only four cases have been reported, and all four are preterm neonates born before 34 weeks of gestation (9). Plasma activities of coagulation factors in preterm neonates are reduced and the fibrinolysis system shows reduced plasminogen activity and increased plasminogen activator inhibitor activity. Furthermore, the presence of UVV could be a predisposition for thrombosis, resulting in the consumption of coagulation factors and the induction of fibrinolysis (9, 10). Premature neonates could be more susceptible to thrombosis and coagulopathy. In our case, no thrombi were observed during pregnancy, however, a turbulent flow in color Doppler was observed. We identified thrombi indicated by a hyperechoic region in the dilated umbilical portion after birth. Thus, we speculated that the combination of UVV and prematurity contributed to DIC after birth.

In the current case, the placental pathologic diagnosis was FTV. It is associated with umbilical anomalies, such as dilation of the umbilical portion and nuchal cord and increased umbilical length. Microthrombi caused by congestion of the fetal or umbilical circulation results in a formation of an embolus in the placenta, which comprises one of the histological characteristics of FTV. The clinical course of FTV varies from being asymptomatic to FGR and IUFD (11). Although FTV is associated with umbilical cord anomalies, no previous case reports of UVV have reported FTV. Furthermore, only a few of the case reports included placental pathology; thus, FTV may be underestimated in cases of UVV. Isolated UVV cases with neonatal complications may benefit from pathological findings from the placenta to understand the pathophysiology of the complications.

Interestingly, FTV has been reported to cause neonatal liver diseases. Two reported cases of FTV resulted in neonatal acute liver failure. Both cases also presented with hyperbilirubinemia, coagulopathy, and cholestasis. One patient was full-term, and the other was a preterm infant. Liver biopsies of these cases showed cholestasis, portal fibrosis, and bile duct proliferation, suggestive of neonatal hepatitis (12). Neonatal hepatitis is usually caused by biliary congestion, and most cases are idiopathic. Patients with idiopathic neonatal hepatitis typically recover fully and rarely require long-term interventions. No evidence of infection, inborn errors, or extrahepatic cholestasis was present in the case; thus, the patient was diagnosed with idiopathic neonatal hepatitis. Although we did not perform a liver biopsy, the elevation of total bile acid and biomarkers for liver fibrosis may readily imply a similar pathogenesis of hepatitis. In this case, obstruction of blood circulation is secondary to FTV, compromising liver circulation, and causing improper liver maturation. In addition, the infant was SGA, indicating FGR, which is also associated with under-perfusion to the liver to shunt the circulation to the brain and heart (13). Therefore, both FGR and FTV can predispose to neonatal liver injury, resulting in hyperbilirubinemia and coagulopathy after birth. Although previous reports on FTV with neonatal hepatitis indicated the hyperbilirubinemia due to hepatitis after birth, in the present case, hyperbilirubinemia occurred within a few hours of life and may have been triggered by polycythemia and hemolysis, as seen with elevated indirect bilirubin, AST, and LD levels. Furthermore, the signs of liver injury did not become apparent until approximately 1 month after birth. Adult autopsies have shown that microthrombi in the portal vein cause liver damage and accelerate liver fibrosis (14). In the present case, we observed a dilated portion of the umbilical vein with turbulent blood flow, suggestive of a thrombotic state. In addition, the D-dimer levels decreased and platelet count increased as a response to FFP and platelet transfusions; however, the slight elevation of D-dimer and low levels of fibrinogen persisted until 34th day of life. The elevation of liver enzymes started at the same time as the resolution of the umbilical cord dilation. Thus, we suspect that chronic blood flow stasis in the umbilical portion, in addition to FTV and FGR, and increased vulnerability to thrombosis, causing neonatal hepatitis.

Regarding polycythemia, it has been reported that infants who are SGA are at a higher risk for complications, such as polycythemia (15, 16). However, there have been no reports of fetal UVV causing polycythemia, and it is unclear whether polycythemia is solely caused by SGA. Furthermore, at present, the association between ROP and UVV is unclear. In the study investigating risk factors associated with the development and progression of ROP, the development and progression of ROP were associated with lower gestational age, and the development of ROP was associated with RDS (17). The study also included potential risk factors such as DIC and blood transfusions, which were not found to be associated risk factors. Thus, we consider aggressive ROP observed in the present case was due to prematurity and the use of oxygen for RDS.

**Figure 4:**
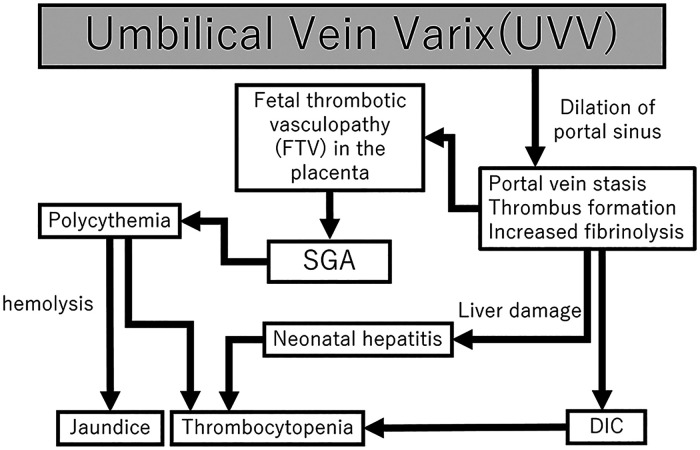
Flowchart of the mechanisms of DIC, polycythemia, and neonatal hepatitis following fetal UVV. Fetal UVV can result in DIC, SGA, and neonatal hepatitis through various mechanisms. DIC, disseminated intravascular coagulation; SGA, small for gestational age; UVV, umbilical vein varix.

We report a case of an ELBWI SGA with isolated UVV and FTV, who also developed DIC, polycythemia, and neonatal hepatitis. It is difficult to explain the etiology of all complications in one scheme. However, all the factors can be explained by the neonatal outcomes (Figure 4). Antenatally, the presence of UVV contributed to FTV, which in turn caused FGR, thrombosis, and hepatic injury. Such predispositions and prematurity, together with low coagulation activities, caused neonatal DIC and hyperbilirubinemia. Furthermore, persistent blood flow stasis in the umbilical portion gave rise to late-onset neonatal hepatitis. Therefore, we speculate that the presence of UVV causes serial antenatal and neonatal pathophysiological changes. Neonatal outcomes associated with isolated UVV in premature infants have been shown to have favorable outcomes without complications. However, only one previous case of ELBWI with isolated UVV has been reported, wherein the infant also experienced neonatal coagulopathy but ultimately survived (9). Notably, ELBWI may be more susceptible to complications due to their prematurity or other factors, such as the location of the UVV and the length of time between detection of UVV and birth. Previous studies have suggested that UVV diagnosed before 26 weeks of gestation is associated with an increased incidence of adverse outcomes (18). Furthermore, FGR may be a sign of neonatal complications due to prolonged fetal distress as opposed to brain-sparing effects and non-reassuring fetal status. Aside from the thrombus formation in UVV, the reason for early termination of pregnancy may also be associated with neonatal outcomes. Further studies on antenatal UVV detection and premature babies are expected. In conclusion, despite the favorable outcome of isolated UVV reported in the literature, UVV with ELBWI and FGR may develop DIC and neonatal hepatitis, requiring extensive management and observation. Further studies of placental pathology in UVV cases are essential to understand its various clinical outcomes.

## Data Availability

The raw data supporting the conclusions of this article will be made available by the authors, without undue reservation.
